# TSCRE: a comprehensive database for tumor-specific *cis*-regulatory elements

**DOI:** 10.1093/narcan/zcad063

**Published:** 2024-01-11

**Authors:** Guanjie Peng, Bingyuan Liu, Mohan Zheng, Luowanyue Zhang, Huiqin Li, Mengni Liu, Yuan Liang, Tianjian Chen, Xiaotong Luo, Xianping Shi, Jian Ren, Yueyuan Zheng

**Affiliations:** Clinical Big Data Research Center, Scientific Research Center, The Seventh Affiliated Hospital of Sun Yat-sen University, Shenzhen 518107, P.R. China; State Key Laboratory of Oncology in South China, Cancer Center, Collaborative Innovation Center for Cancer Medicine, School of Life Sciences, Sun Yat-sen University, Guangzhou 510060, China; Guangzhou Municipal and Guangdong Provincial Key Laboratory of Protein Modification and Degradation, Affiliated Cancer Hospital of Guangzhou Medical University, State Key Laboratory of Respiratory Disease, School of Basic Medical Sciences, Guangzhou Medical University, Guangzhou 510120, China; Clinical Big Data Research Center, Scientific Research Center, The Seventh Affiliated Hospital of Sun Yat-sen University, Shenzhen 518107, P.R. China; State Key Laboratory of Oncology in South China, Cancer Center, Collaborative Innovation Center for Cancer Medicine, School of Life Sciences, Sun Yat-sen University, Guangzhou 510060, China; Guangzhou Municipal and Guangdong Provincial Key Laboratory of Protein Modification and Degradation, Affiliated Cancer Hospital of Guangzhou Medical University, State Key Laboratory of Respiratory Disease, School of Basic Medical Sciences, Guangzhou Medical University, Guangzhou 510120, China; State Key Laboratory of Oncology in South China, Cancer Center, Collaborative Innovation Center for Cancer Medicine, School of Life Sciences, Sun Yat-sen University, Guangzhou 510060, China; State Key Laboratory of Oncology in South China, Cancer Center, Collaborative Innovation Center for Cancer Medicine, School of Life Sciences, Sun Yat-sen University, Guangzhou 510060, China; State Key Laboratory of Oncology in South China, Cancer Center, Collaborative Innovation Center for Cancer Medicine, School of Life Sciences, Sun Yat-sen University, Guangzhou 510060, China; Clinical Big Data Research Center, Scientific Research Center, The Seventh Affiliated Hospital of Sun Yat-sen University, Shenzhen 518107, P.R. China; Clinical Big Data Research Center, Scientific Research Center, The Seventh Affiliated Hospital of Sun Yat-sen University, Shenzhen 518107, P.R. China; State Key Laboratory of Oncology in South China, Cancer Center, Collaborative Innovation Center for Cancer Medicine, School of Life Sciences, Sun Yat-sen University, Guangzhou 510060, China; Guangdong Institute of Gastroenterology, Department of General Surgery, Guangdong Provincial Key Laboratory of Colorectal and Pelvic Floor Diseases, The Sixth Affiliated Hospital, Sun Yat-sen University, Guangzhou 510060, China; Guangzhou Municipal and Guangdong Provincial Key Laboratory of Protein Modification and Degradation, Affiliated Cancer Hospital of Guangzhou Medical University, State Key Laboratory of Respiratory Disease, School of Basic Medical Sciences, Guangzhou Medical University, Guangzhou 510120, China; State Key Laboratory of Oncology in South China, Cancer Center, Collaborative Innovation Center for Cancer Medicine, School of Life Sciences, Sun Yat-sen University, Guangzhou 510060, China; Clinical Big Data Research Center, Scientific Research Center, The Seventh Affiliated Hospital of Sun Yat-sen University, Shenzhen 518107, P.R. China

## Abstract

*Cis*-regulatory elements (CREs) and super *cis-*regulatory elements (SCREs) are non-coding DNA regions which influence the transcription of nearby genes and play critical roles in development. Dysregulated CRE and SCRE activities have been reported to alter the expression of oncogenes and tumor suppressors, thereby regulating cancer hallmarks. To address the strong need for a comprehensive catalogue of dysregulated CREs and SCREs in human cancers, we present TSCRE (http://tscre.zsqylab.com/), an open resource providing tumor-specific and cell type-specific CREs and SCREs derived from the re-analysis of publicly available histone modification profiles. Currently, TSCRE contains 1 864 941 dysregulated CREs and 68 253 dysregulated SCREs identified from 1366 human patient samples spanning 17 different cancer types and 9 histone marks. Over 95% of these elements have been validated in public resources. TSCRE offers comprehensive annotations for each element, including associated genes, expression patterns, clinical prognosis, somatic mutations, transcript factor binding sites, cancer-type specificity, and drug response. Additionally, TSCRE integrates pathway and transcript factor enrichment analyses for each study, enabling in-depth functional and mechanistic investigations. Furthermore, TSCRE provides an interactive interface for users to explore any CRE and SCRE of interest. We believe TSCRE will be a highly valuable platform for the community to discover candidate cancer biomarkers.

## Graphical Abstract

**Graphical Abstract:**
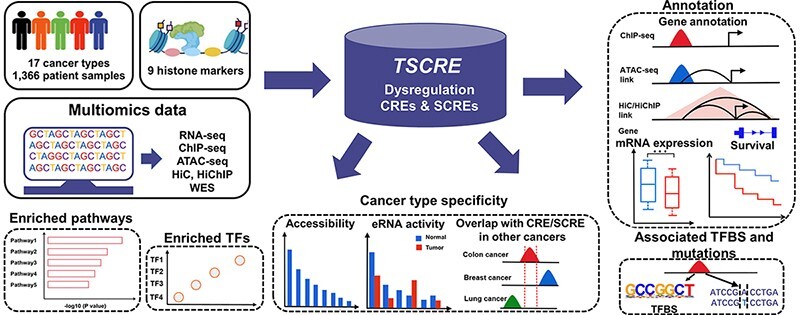

## Introduction

*Cis*-regulatory elements (CREs), including enhancers, promoters, and silencers, are crucial for controlling gene expression during development (1–3). Super *cis-*regulatory elements (SCREs), characterized by large clusters of regulatory regions, are essential in determining cell differentiation and identity (4–6). Numerous CREs and SCREs have been identified across human tissues and diseases. In the context of human cancers, the aberrant activity of CREs and SCREs can lead to the dysregulation of oncogenes and tumor suppressors, disrupting normal cellular processes and promoting cancer hallmarks such as proliferation, invasion and metastasis (7–9). Dysregulations in enhancers, super-enhancers, silencers, and promoters are common epigenetic alterations. For example, aberrant regulation of enhancers and super-enhancers have been frequently observed in various cancers, resulting in the upregulation of key oncogenes, such as *MYC* and *FOXQ1* in colorectal cancer (10), as well as *FOXC1* and *MET* in triple-negative breast cancer (11). Additionally, many silencers, characterized by extensive broad H3K27me3 modification, exhibit a significant reduction in H3K27me3 modification size in human tumors, thereby promoting the activation of oncogenes such as *MYH11* and *EGFR* (12). Moreover, dysregulation of promoters, including promoter hypermethylation and alterations in H3K4me3 along with H3K27ac, is frequently observed in cancer (13,14). These dysregulations of CREs and SCREs exhibit specificity towards particular cancer types or subtypes, reflecting the unique epigenomic landscapes of different cells and tumors (15,16). Recent studies have highlighted the potential of dysregulated CREs and SCREs as promising targets for cancer diagnosis and treatment (17,18). For instance, the exclusive activity of the *INSM1* promoter in insulinoma tumors has been leveraged for adenoviral therapy in insulinoma treatment (19). Therefore, the accumulating evidence for the essential roles of dysregulated CREs and SCREs in cancer biology, emphasizes the urgent need to comprehensively catalog dysregulated CREs and SCREs across various human cancer types.

Previous studies have demonstrated the efficiency and robustness of histone modification profiling in identifying distinct classes of regulatory elements (20–22). For example, promoters can be identified by the histone mark H3K4me3, enhancers by H3K4me1, active regulatory elements by H3K27ac, and repressive elements by either H3K27me3 or H3K9me3 (18). With the rapid accumulation of chromatin immunoprecipitation sequencing (ChIP-seq) data on histone marks, several CRE and SCRE databases have been developed, such as ENCODE (23), CistromeDB (24), ChIP-Atlas (25), SEdb v2.0 (26), SEA v3.0 (27) and dbSUPER (28). These databases serve as valuable resources for investigating *cis-*regulatory elements, but they primarily focus on profiling of CREs or SCREs within individual tissues and cells. There is an urgent need for the collection of feature-specific dysregulated elements in human cancers (e.g. tumor-specific, metastasis-specific, and subtype-specific elements). Additionally, besides super-enhancers, other broad regulatory elements, such as super repressive elements and broad H3K4me3 regions, have also been implicated in cancer (6,9,11,25,26), emphasizing the importance of their efficient identification and characterization. Moreover, further research on dysregulated CREs and SCREs heavily relies on reliable regulatory annotation, including genetic and epigenetic annotation, as well as association analyses involving transcription factors, cancer pathways, clinical prognosis, and response to anticancer drugs. Therefore, it is necessary to develop a comprehensive database dedicated to cancer-associated CREs and SCREs, elucidating their regulatory mechanisms in a highly cancer-specific context.

**Figure 1.**
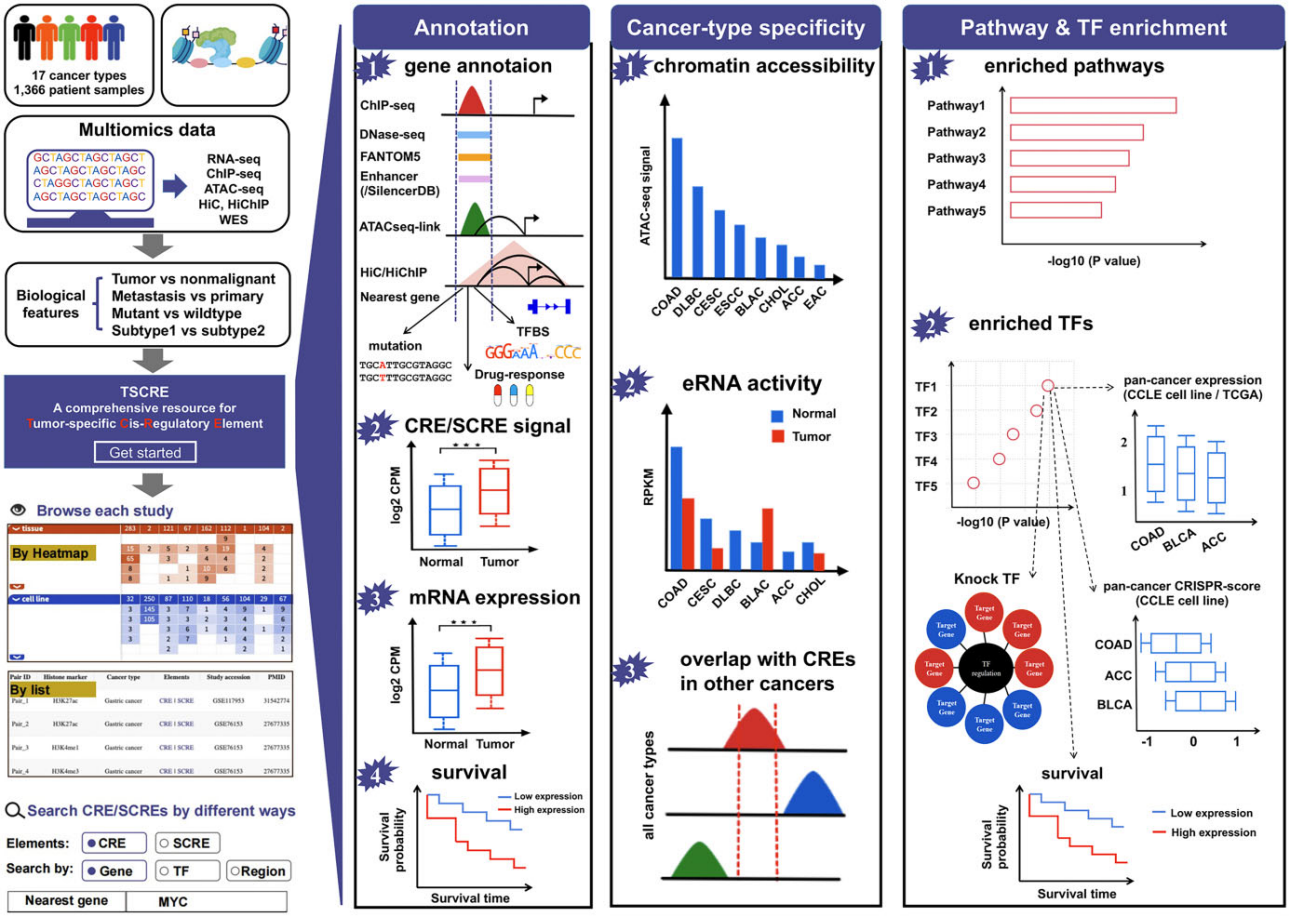
Overall design and construction of TSCRE.

Here, we present TSCRE (http://tscre.zsqylab.com/), a comprehensive open resource of tumor-specific and cell type-specific CREs and SCREs derived through extensive re-analyses of public histone modification profiling data in human cancers (Figure 1, Supplementary Table S1). Using TSCRE, users are able to efficiently and intuitively explore dysregulated CREs or SCREs of interest in various cancer types of contrast experiments, such as ‘tumor vs. nonmalignant’, ‘metastasis vs. primary’, "mutant vs. wildtype’. We believe this platform will greatly benefit the research community by aiding in the screening of candidate CREs and SCREs, as well as facilitating the identification of relevant transcriptional regulators in human cancers.

## Materials and methods

### Data collection

A systematic search was conducted to find out cancer-associated histone modification profiles. Firstly, the GEO and SRA database were searched using keywords relating to nine of better-known histone markers (including H3K27ac, H3K4me3, H3K4me2, H3K4me1, H3K36me3, H3K9ac, H3K79me2, H3K9me3 and H3K27me3), along with the term ‘Genome binding/occupancy profiling by high throughput sequencing’. Datasets were restricted to human studies published before February 2023. Then we performed a manual screening and reserved studies that met the following criteria: 1) inclusion of tumor tissue or cancer cell lines, 2) samples in each study that could be compared based on a specific biological condition, including ‘tumor vs. non-malignant,’ ‘metastasis vs. primary,’ ‘mutant vs. wild-type’ or ‘subtype1 vs. subtype2’ and 3) presence of at least two biological replicates for each condition. In addition, matched RNA-seq data from the corresponding studies were downloaded when available.

The following additional datasets were collected: long-range interactions between genes and distal regulatory regions, including pan-cancer ‘enhancer-to-gene’ links from TCGA ATAC-seq projects (29), Hi-C loops from 3D Genome Browser (30), and HiChIP loops from matched studies. Other *cis-*regulatory elements were obtained from public resources, containing DNase I-hypersensitive sites (DHSs) from the ENCODE project (23), CREs from the FANTOM5 project (31), enhancers from GeneHancer (32), silencers from SilencerDB (33) and super-enhancers from a recent publication (34). Multi-omics data of pan-cancer tissues and cell lines were extracted from TCGA and DepMap projects, containing mRNA expression, somatic mutations, chromatin accessibility, clinical data and CRISPR scores (35,36). In addition, we also obtained enhancer RNA (eRNA) expression from the TCeA Portal and mutations from COSMIC (37,38). TFBSs from the ENCODE project and TF target genes from KnockTF were also included in our database (39). Lastly, we acquired pharmacogenomic data from GDSC2 and CTRP2, which involved drug response data from 198 and 545 compounds in various cancer lineages (40,41).

### Data preprocessing

Raw ChIP-seq reads were trimmed using Trim-galore (version 0.6.6) and then aligned to the GRCh38 genome (ENSEMBL release 84) using BWA (version 0.7.17) with default parameters (42). Then uniquely mapped reads were extracted and sorted using SAMtools (version 1.7) program with the ‘-q 1’ option (43). PCR duplicates were removed using the Picard MarkDuplicates tool (version 2.26.2), and ENCODE blacklist regions were excluded using BEDtools (version v2.26.0) (44). MACS2 (Model-Based Analysis of ChIP-Seq, version 2.2.6) was applied to call peaks (refer to CREs) with the options ‘-q 0.01 –extsize = 146 –nomodel’ (45). Samples meeting all the following criteria are retained for further analysis: (i) duplication rates <0.5; (ii) mapping rates >0.6; (iii) at least 5 million usable fragments for narrow marks and 15 million for broad marks; (iv) peaks >500 and fraction of reads in peaks (FRiP) ≥1%.

Similarly, raw RNA-seq reads were processed to remove low-quality reads using Trim-galore (version 0.6.6) with default parameters. High-quality reads were then aligned to the GRCh38 genome using HISAT2 (version 7.2.0) and gene quantification was performed using the htseq-count program (version 0.11.3) with default settings (46,47). Samples with a mapping rate >0.6 and at least 10 million usable fragments were selected for differential gene expression analysis. The DESeq2 package or Wilcoxon test (≥8 samples in each given condition) were applied to identify differentially expressed genes, employing the criteria of ‘adjusted *P*-value <0.05 and absolute fold-change >1.5’ (48).

### Dysregulated CRE/SCRE identification

Peaks modified by different histone markers, as identified in the previous section, were considered as CREs. DiffBind (version 3.6.5) was then used to compare CREs between two conditions and those with a fold change ≥1.5 and FDR <0.05 were selected as dysregulated CREs (49).

Among SCREs, the most extensively studied subset are super-enhancers, usually marked by H3K27ac modification. Rank Order of Super Enhancers (ROSE) is a commonly used method for identifying super-enhancers (5). Briefly, ROSE merged enhancer elements within a 12.5-kb distance and arranges them in decreasing order of intensities. Super-enhancers are then defined as stitching elements exhibiting a tangent slope with an inflection point value ≥1. ROSE (version 1.0) was also applied to identify super repressive elements enriched with H3K27me3 modification (6). Considering the potential variations in the stitching distance for different histone marks, we initially determined the most suitable distance for each histone mark by analyzing the histone modification peaks across various cell types from the ENCODE project. We merged peaks within different stitching distances for each dataset, ranging from 0.5 to 24 kb with 0.5 kb increments, and identified SCREs at each distance. We then counted the total number of peaks contained in SCREs and selected the optimal distance when the increasing number at that distance stabilized compared to the previous distance. As shown in Supplementary Figure S1, H3K27ac and H3K27me3 peaks within SCREs reached stability at 12 and 5 kb, respectively, which were close to the reported values of 12.5 and 4 kb. After obtaining the optimal distance for each histone marker (Supplementary Figure S1), we used ROSE to identify SCREs for each sample. Next, we employed the BEDtools multiinter function to find a consensus SCRE set of unique genomic intervals presenting in at least two samples for each study. Finally, DiffBind program was applied to identify the dysregulated SCREs and those with a fold change ≥1.5 and *P* value <0.05 were selected.

### Annotations of dysregulated CRE/SCREs

We validated the accuracy of CREs and SCREs by checking whether they overlapped with regulatory regions obtained from ENCODE, FANTOM5, GeneHancer, SilencerDB and other existing literature (34). We then applied three gene annotation strategies to find the associated genes for each element. First, we mapped CREs to the nearest genes using the Homer annotatePeaks.pl function (50), while mapping SCREs to the nearest or overlapping genes with the help of ROSE_geneMapper.py function. Second, we associated distal CREs and SCREs with ATAC-seq-linked genes, which were established by the TCGA consortium based on the correlation between ATAC-Seq peaks and the expression levels of neighboring genes. Lastly, we assigned distal CREs and SCREs to their respective genes using Hi-C and HiChIP loops. To assist researchers in identifying the most relevant regulatory genes, we calculated mRNA expression level for each associated gene from available matched samples, pan-cancer tissues as well as cancer cell lines, and examine the survival outcome in cancer samples. Furthermore, associated TFBSs and somatic mutations were assigned to each CRE and SCRE using the BEDTools intersect program.

### Assessing cancer-type specificity and examining associations with drug response

The cancer-type specificity of each regulatory element was determined by calculating its overlap with other elements in TSCRE, as well as by assessing the chromatin accessibility and enhancer RNA activity in the TCGA pan-cancer landscape. Additionally, we employed calcPhenotype function from the oncoPredict package to impute the drug response from GDSC and CTRP cancer cell lines to TCGA patient samples, after which we calculated the associations of each regulatory element with the imputed drug response (51). To achieve this, we trained linear ridge regression models using the expression levels and drug sensitivity scores of the cancer cell lines from GDSC and CTRP. These models were then applied to the expression levels of TCGA samples, generating predicted scores for drug sensitivity for each TCGA patient. Next, we evaluated the accessibility of each regulatory element in the corresponding TCGA patients using TCGA ATAC-seq signals, and further determined the associations between regulatory element accessibility and imputed drug response using Spearman correlation. Associations with |*R*| >0.3 and FDR <0.05 were considered as significant associations in each cancer type.

### Identification of enriched TFs and biological functions

Enrichment analysis of TF binding sites was conducted for dysregulated elements using the Homer findMotifs.pl function and LOLA program, setting one condition's dysregulated CRE/SCREs as the foreground and the other's as the background (52). To gain a deeper understanding of the context specificity and essentiality of each significant TF (FDR < 0.01), we analyzed their corresponding expression levels in TCGA pan-cancer samples, gene dependency scores in cancer cell lines, and clinical prognosis. Furthermore, we introduced the KnockTF datasets and presented the potential downstream targets.

For biological functions, we employed the solo mode of Cistrome-GO program to perform pathway enrichment analysis by using the collections of gene sets from KEGG and GO-BP (53). A minimum-hypergeometric test was conducted to identify the enriched pathways with an FDR < 0.2. The detailed information about association analyses were listed in Supplementary Table S2.

### Identification of tumor-specific and cell type-specific CREs and SCREs

To identify more CREs and SCREs that regulate oncogenes in tumor-specific and cell type-specific manners, we first selected the CREs and SCREs that demonstrated gained active modification or lost repressive modification in tumor samples compared with nonmalignant samples. We then employed a prioritization approach to select the most promising candidates from all association analyses based on the specific criteria. These criteria included: (i) enhanced expression of the associated gene, (ii) ranking within the top 5 for chromatin accessibility, (iii) ranking within the top 5 for eRNA activity and (iv) exhibiting an overlap of at least 50% in length with fewer than two *cis-*regulatory elements found in other cancer types. CREs and SCREs that met all these criteria were considered to have a high confidence level of tumor specificity and cancer-type specificity. Additionally, CREs and SCREs that satisfied three criteria achieved a median confidence level. Furthermore, CREs and SCREs that met two criteria were regarded as having a low confidence level.

### Web interface implementation

All the metadata and analysis results were stored and managed using MySQL tables. The web interfaces were implemented using Hyper Text Markup Language (HTML), Cascading Style Sheets (CSS), and JavaScript (JS). To visualize all the analysis results, various statistical diagrams were shown by EChars and UCSC Genome Browser were implemented.

## Results

### Data summary

In the current release, TSCRE contains 138 carefully curated histone ChIP-seq datasets covering 1366 patient samples across 9 different histone marks and 17 different cancer types (Supplementary Table S1). Of these, 1034 samples were collected from patient tissues, while 332 samples were derived from cancer cell lines (Supplementary Table S1C). Across 17 cancer types, a total of 18 64 941 dysregulated CREs and 68 253 dysregulated SCREs were identified in one of the following conditions: ‘tumor versus nonmalignant’, ‘metastasis versus primary’, ‘mutant versus wildtype’ and ‘subtype1 versus subtype2’ (Table 1). Notably, approximately 95% of CREs and 99% of SCREs have been validated in at least one public resource, demonstrating the reproducibility and robustness of our data analyses.

**Table 1.** Statistics of dysregulated CREs and SCREs in TSCRE

	Tumor versus nonmalignant		Metastasis versus primary		Mutant versus wildtype		Subtype1 versus Subtype2	
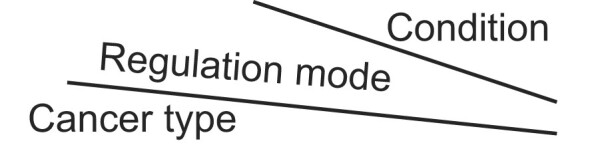	CRE	SCRE	CRE	SCRE	CRE	SCRE	CRE	SCRE
Colorectal cancer	143 662	4878	−	−	−	−	−	−
Uterine leiomyoma	68 805	2481	−	−	−	−	−	−
Gastric cancer	58 686	2339	−	−	−	−	−	−
Prostate cancer	40 833	2422	68805	2170	−	−	−	−
Liver cancer	32 847	1187	−	−	−	−	−	−
Kidney cancer	32 209	3000	8	98	−	−	341 735	8502
Glioma	18 657	1481	−	−	−	−	−	−
Esophageal cancer	7841	442	2145	172	−	−	20 562	560
Follicular lymphoma	257	170	−	−	−	−	−	−
Diffuse large B cell lymphoma	339	82	−	−	−	−	−	−
Natural killer T-cell lymphoma	27 784	713	−	−	−	−	−	−
Lung cancer	25 066	1584	−	−	−	−	−	−
Breast cancer	543 577	17 615	−	−	−	−	296 088	11 537
Head and neck cancer	51 649	3276	−	−	−	−	9796	1052
Pancreatic cancer	7198	81	59 098	1669	−	−	−	−
Osteosarcoma	−	−	193	207	−	−	−	−
Chronic lymphocytic leukemia	−	−	−	−	7101	535	−	−
Total	1 059 410	41 751	130 249	4316	7101	535	668 181	21651

To facilitate further functional and mechanistic studies, systematic association analyses are integrated into TSCRE. First, we provide detailed gene annotation, associated TFBSs and somatic mutations for each dysregulated element. In addition to the nearest or overlapping genes, a total of 220 681 distal CREs and 44 639 distal SCREs were associated with specific genes through distal element-to-promoter loops. Additionally, 80.09% (1 493 595/1 864 941) of CREs and 99.96% (68 224/68 253) of SCREs exhibited one or more TFBSs. Moreover, 31 705 dysregulated CREs and 27 029 dysregulated SCREs had associated somatic mutations in the corresponding cancer type. These findings highlight the importance of *cis-*regulatory elements in addressing key issues related to cancer biology. Second, we accessed the cancer-type specificity of each regulatory element. Notably, a large proportion of CREs (83.8%) and SCREs (59.58%) show overlap with other regulatory elements in three or fewer cancer types, indicating a high degree of cancer-type specificity (Supplementary Figure S2). Third, we assessed the association between regulatory elements and imputed drug response in patients across various cancer types, providing a more direct and powerful evaluation of the role of CREs and SCREs in targeted therapy compared to *in vitro* cell lines. We obtained 20 812 864 associations between CRE accessibility and the imputed drug response, consisting of 6 531 763 associations detected from CTRP and 14 281 101 associations from GDSC. Similarly, 672 248 associations were found in SCREs. These data provide valuable insights into the potential impact of regulatory elements on drug response in cancer patients. The dysregulation of CRE and SCREs can influence transcriptional programs by facilitating or restricting the accessibility of transcript factor binding sites in a tumor-specific and cell type-specific manner, thereby mediating the activation of cancer-associated pathways. We further identified all enriched TFs and pathways for each study to promote follow-up functional and mechanistic studies. Additionally, we collected a comprehensive dataset comprising 683 TF knockout microarrays and RNA-seq data from the KnockTF database, enabling the identification of potential downstream targets.

### Data access

**Figure 2.**
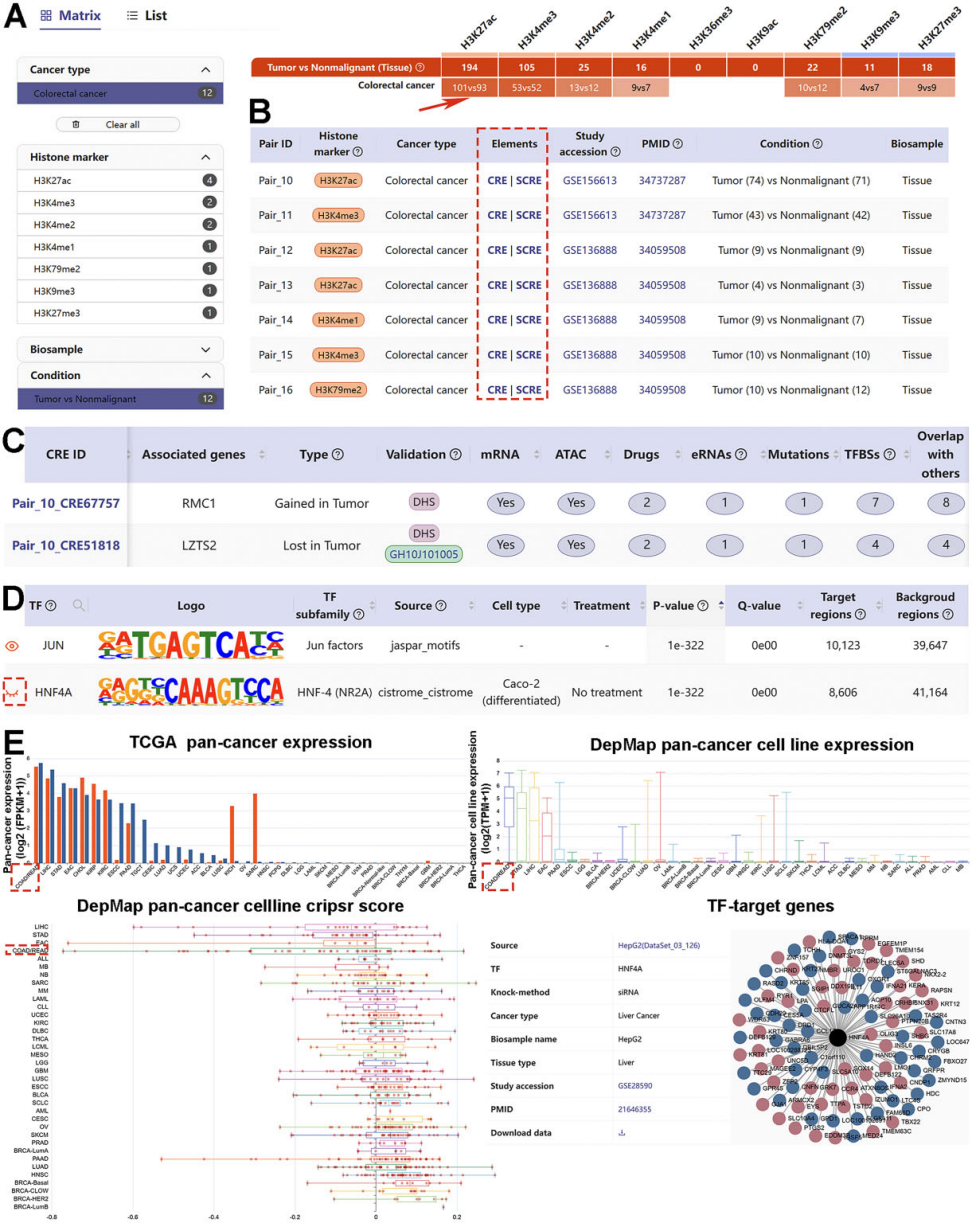
A schematic workflow of the browse interface in TSCRE. **(A)** TSCRE provides an interactive heatmap and a summary list to browse and select studies of interest. Snapshot of results for colorectal cancer in ‘Tumor vs Nonmalignant’ condition. **(B)** A summary list is shown when clicking on a specific heatmap cell. Data in TSCRE is organized into two layers: CRE and SCRE. **(C)** The CRE information section shows all dysregulated elements and summarizes the number of association analyses conducted for each element. **(D)** Snapshot of HNF4A as the second most enriched TF. **(E)** Click the ‘eye’ button to get the expression levels of HNF4A in pan-cancer tissues and cell lines, the gene dependency scores, as well as potential target genes.

TSCRE provides a user-friendly web interface, allowing users to intuitively explore and search any CRE or SCRE:

**Explore**. Users can select the cancer type(s), histone marker(s) and condition(s) of interest either through an interactive heatmap or a summary list. For example, if users are interested in studying tumor-specific regulatory elements in colorectal cancer, they can easily choose ‘colorectal cancer’ and ‘Tumor vs Nonmalignant’ from the metadata selection facet (Figure 2A). Subsequently, the relevant heatmap cells are retrieved (Figure 2A), and upon clicking on a specific cell, a dataset list is presented (Figure 2B). Data in TSCRE is organized into two layers: CRE and SCRE. By clicking on ‘CRE’ or ‘SCRE’ in a dataset of interest, users will be directed to a detailed page that provides study and sample details, CRE/SCRE information, as well as TF and pathway enrichment analysis. The CRE/SCRE information section shows all dysregulated elements and summarizes the number of association analyses conducted for each element (Figure 2C). Clicking on individual CRE ID shows the detailed information for each dysregulated element. The TF enrichment analysis section displays significantly enriched TFs identified in this study (Figure 2D). Some visualized figures are generated in real time to allow investigation of the cancer-type specificity and potential targets of enriched TFs (Figure 2E). For instance, HNF4A, a well-established gastrointestinal-specific transcription factor (54), ranked as the second most enriched TF in colorectal cancer-specific CREs (Figure 2D). HNF4A exhibited the highest expression levels in both colorectal cancer tissues and cell lines, regulated many target genes and was essential for the viability of the majority of colorectal cancer cells in the unbiased high-throughput CRISPR screening (Figure 2E). The pathway enrichment analysis section displays all enriched pathways associated with dysregulated elements and the top 10 pathways are shown in a bubble plot.

**Search**. TSCRE provides three modes to query the database, according to your interest in specific genes, transcription factors, and genomic regions. All the search results are summarized with real-time statistical diagrams and further filtered by check boxes.

### Tumor-specific CREs and SCREs identified in TSCRE

**Figure 3.**
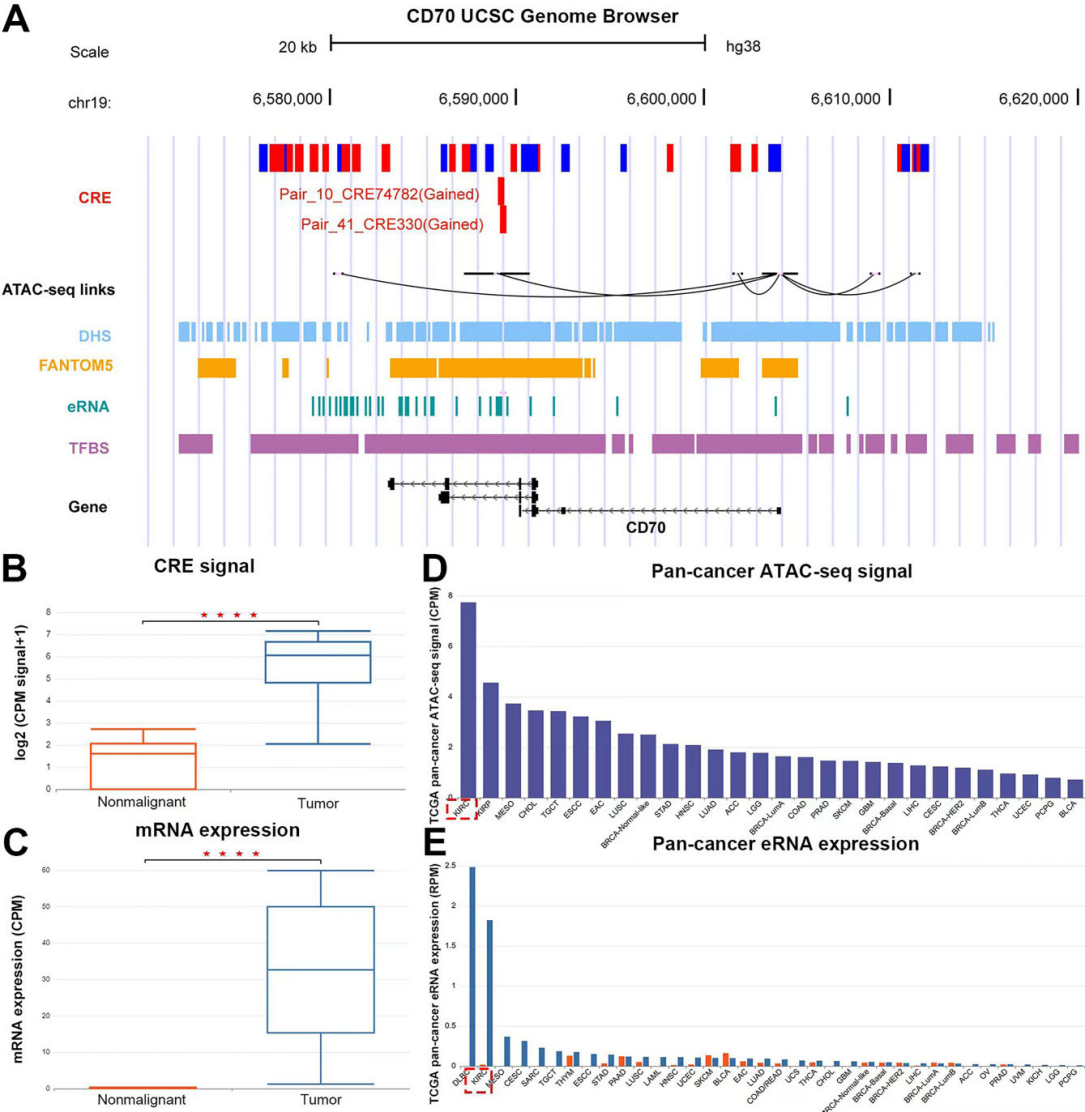
The detailed information of a distal CRE for *CD70*. **(A)** Visualization of ‘Pair_41_CRE330’ using UCSC Genome Browser. (B, C) A significant increase in H3K27ac signal **(B)** and mRNA expression **(C)** was observed in kidney cancer. (D, E) Kidney cancer exhibits the highest chromatin accessibility **(D)** and the second-highest level of eRNA activity **(E)** within this CRE.

TSCRE is a valuable resource to explore and discover cancer biomarkers. Through the analysis of the ‘Tumor vs Nonmalignant’ comparison, we identified 918 CREs and 405 SCREs that exhibited a high confidence level of tumor specificity and cancer-type specificity (see Materials and methods). Additionally, 24 821 CREs and 1948 SCREs met the criteria for the median confidence level. Furthermore, we identified 151 273 CREs and 3880 SCREs with a low confidence level (Supplementary Table S3). These *cis-*regulatory elements may aid in the discovery of candidate cancer biomarkers and enhance our knowledge of regulatory mechanisms in cancer. For example, *CD70* is reported as a tumor-specific biomarker in kidney cancer, which promotes immune escape by inducing cytotoxic effects on B and T lymphocytes. Using TSCRE, we found *CD70* was regulated by a distal enhancer (‘Pair_41_CRE330’) in kidney cancer, which were further validated by FANTOME5 enhancers and pan-cancer ATAC-seq links (Figure 3A). Notably, this enhancer exhibits a significant increase in H3K27ac signal in kidney cancer compared to nonmalignant samples (Figure 3B), which coincides with the upregulation of *CD70* (Figure 3C). Moreover, this enhancer demonstrated a high degree of cell type specificity, as it ranked first in chromatin accessibility (Figure 3D) and second in eRNA activity (Figure 3E) within kidney cancer. Especially, only a single CRE in colon cancer exhibits partial overlap with this enhancer (Figure 3A). These findings indicate this enhancer associated with *CD70* regulation is highly specific to kidney cancer. Another notable case is *MERTK* gene, which is known as an oncogene that promotes breast cancer progression (12). Consistently, nonmalignant samples encompass repressive CREs (known as silencers in SilencerDB) associated with *MERTK*. However, across all subtypes of breast cancer, there is a significant loss of H3K27me3 modification, coinciding with the upregulation of MERTK in most subtypes (Supplementary Figure S3). These results suggest that dysregulated CREs in TSCRE are highly specific to tumorigenesis and underscore their potential role in elucidating the molecular mechanisms underlying pathogenesis.

Similarly, dysregulated SCREs are also highly specific to tumorigenesis (Supplementary Figure S3B). For example, *PDX1*, a potential tumor marker in colorectal cancer (55) (Supplementary Figure S4A), contains both a lost repressive SCRE (‘Pair_18_SCRE66’) and a gained active SCRE (‘Pair_10_SCRE127’, a super-enhancer cataloged in Reference (34)) in colorectal cancer. In nonmalignant tissues, *PDX1* is characterized by the presence of H3K27me3 signal and the absence of H3K27ac. In contrast, it undergoes a reduction in H3K27me3 and an increase in H3K27ac during tumorigenesis, accompanied by elevated mRNA expression. Additionally, this SCRE shows high cell type specificity, ranking first in both accessibility and eRNA activity.

In addition, TSCRE serves as a valuable platform for identifying potential therapeutic targets associated with dysregulated elements. An exemplary case is the EGFR gene, which exhibits frequent overexpression in basal breast cancer and contributes to the aggressive behavior of this subtype (56). Recent investigations have explored EGFR inhibitors, such as gefitinib and erlotinib, as potential treatment modalities for basal breast cancer (56). Encouragingly, our findings demonstrate a significant association between the drug response to gefitinib and erlotinib and a gained active SCRE ("Pair_92_SCRE61’, a super-enhancer cataloged in Reference (34)) in EGFR specific to basal breast cancer (Supplementary Figure S5). Remarkably, within TSCRE, a substantial proportion of CREs (628 993 out of 1 864 941) and SCREs (25 964 out of 68 253) are linked to drugs, suggesting their potential involvement in drug response or as therapeutic targets.

## Discussion

TSCRE is a comprehensive open resource for providing dysregulated CREs and SCREs through the re-analysis of publicly available ChIP-seq data. In comparison to other existing databases, TSCRE possesses the following advantages: (i) To the best of our knowledge, TSCRE is the first comprehensive database focusing specifically on *cis-*regulatory elements in a highly cancer-associated context; (ii) TSCRE is the only database emphasizing the roles of both CREs and SCREs. (iii) In addition to super-enhancers, TSCRE also provides super repressive elements and other broad regions, which were modified with repressive marks (e.g. H3K27m3, H3K9me3) or other active marks (e.g. H3K4me3, H3K36me3). (iv) TSCRE provides detailed annotations for each CRE and SCRE, including gene annotation, mRNA expression, clinical prognosis, associated TFBSs, associated mutations and cancer-type specificity. These annotations assist biologists in identifying relevant biological features and discover novel cancer biomarkers. (v) TSCRE assesses the associations between dysregulated elements and drug response in cancer patients, potentially enabling more direct and impactful target therapy. (vi) TSCRE integrates the results of pathway and TF enrichment analysis to facilitate follow-up functional and mechanistic studies.

In the future, TSCRE will be continuously updated as new histone modification profiling data become available in public databases. We also plan to incorporate other epigenetic marks, such as DNA methylation, and more drug response dataset into TSCRE. Additionally, we strongly encourage users to contribute to TSCRE by uploading their own data.

## Supplementary Material

zcad063_Supplemental_Files

## Data Availability

No new data were generated or analysed in support of this research.
