# Supplementary material for: TSCRE: a comprehensive database for tumor-specific cis-regulatory elements
Source: NAR Cancer. 2024 Jan 11;6(1):zcad063. doi: 10.1093/narcan/zcad063 (PMC10782923; doi:10.1093/narcan/zcad063)
Supplement: zcad063_Supplemental_Files [file zcad063_supplemental_files.zip › Supplementary Figure.pdf]

Supplementary Figure

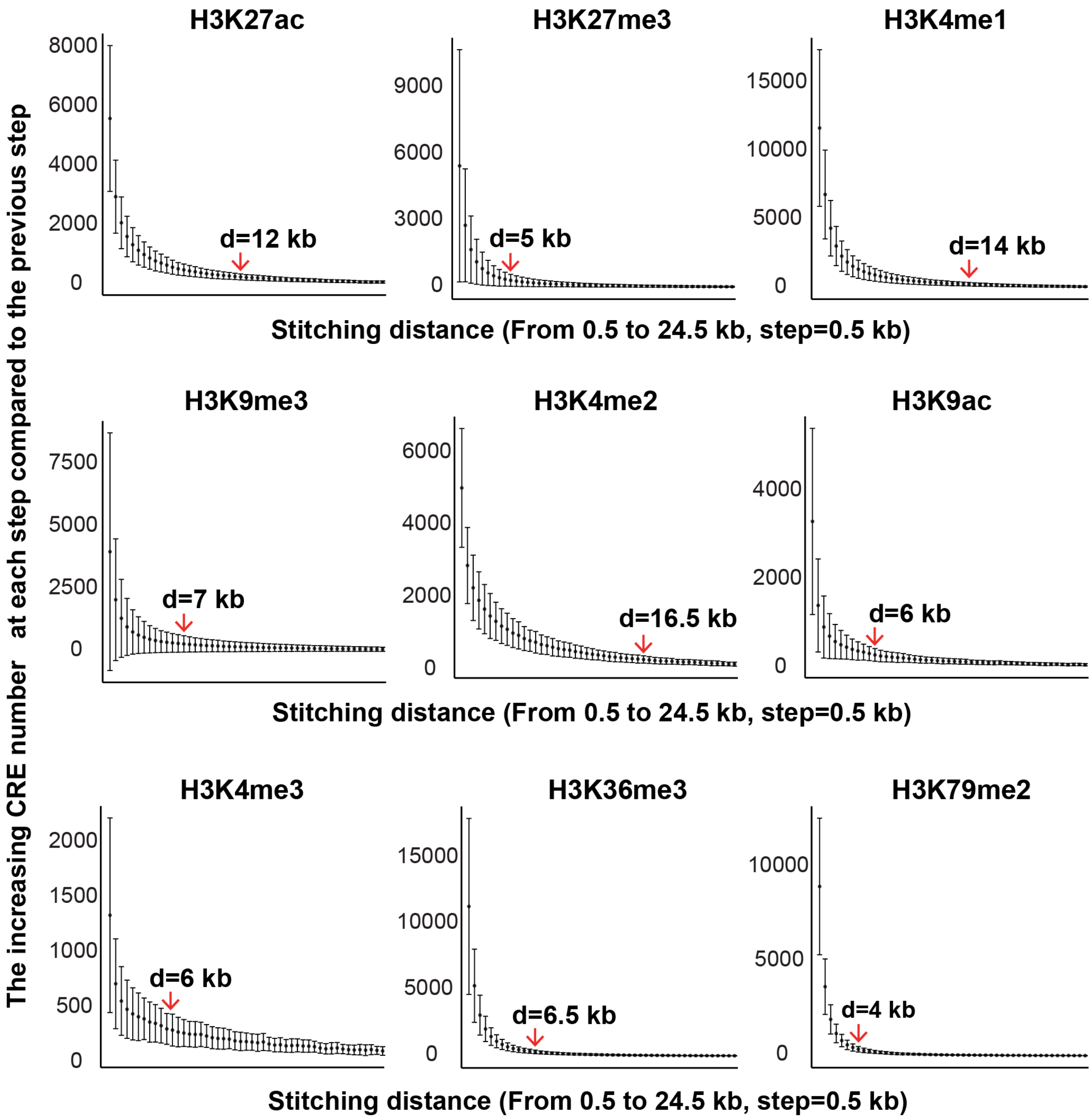

Supplementary Figure 1. The increasing CRE number at each step compared to previous step. We merged peaks within different distances for each dataset, ranging from 0.5 kb to 24 kb with 0.5 kb increments. Then count the total number of peaks

contained in the merged elements and select the optimal distance when the increasing number at that distance stabilized compared to the previous distance.

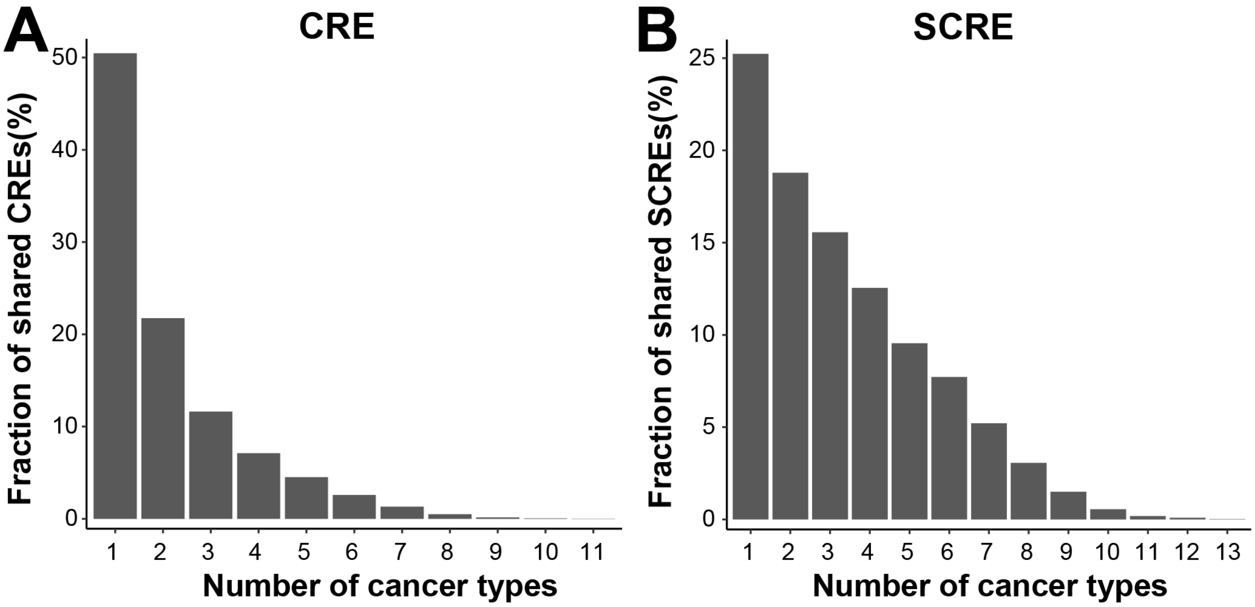

Supplementary Figure 2. A large proportion of dysregulated CREs and SCREs exhibit a high degree of cancer-type specificity. (A) The fraction of shared CREs. **(B)** The fraction of shared SCREs.

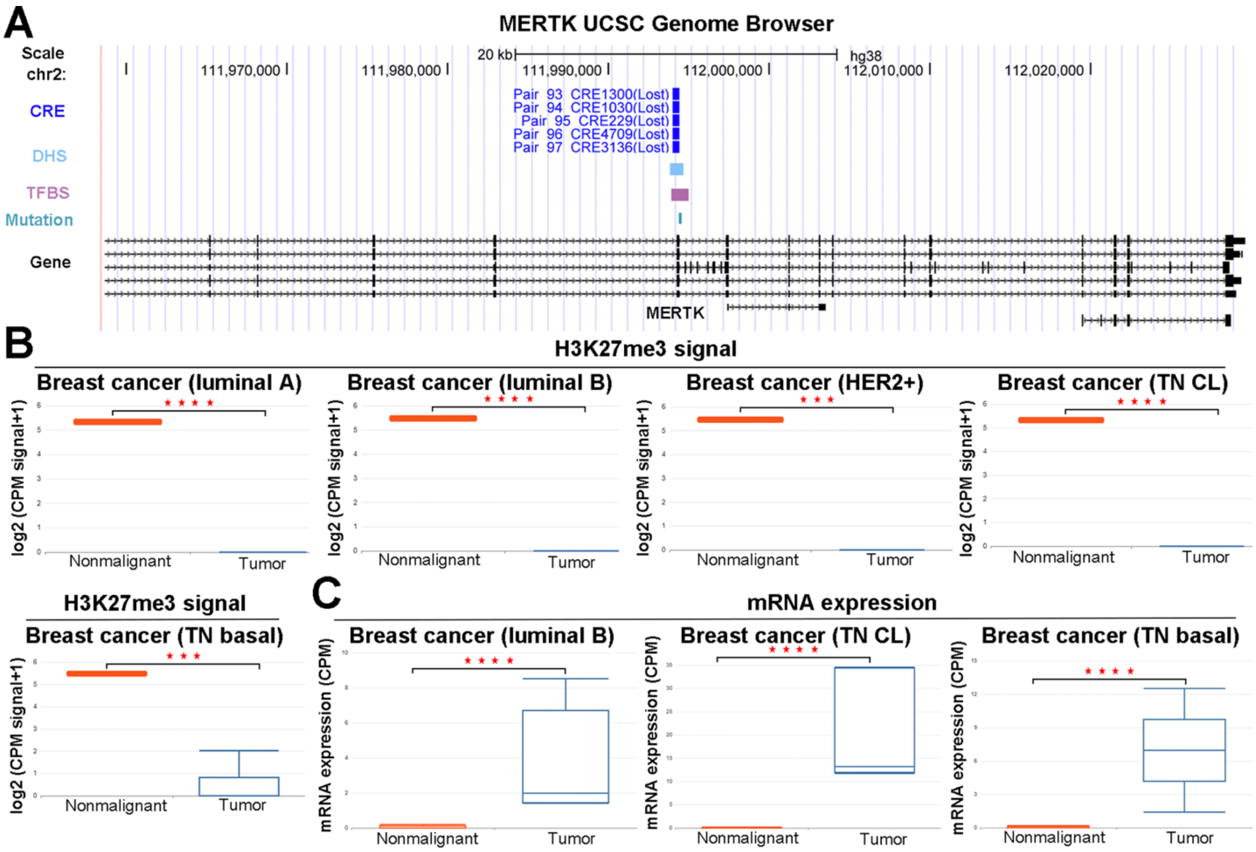

Supplementary Figure 3. An example of repressive CREs for MERTK oncogene. (A) Visualization of repressive CREs using UCSC Genome Browser. **(B)** H3K27me3 modification is selectively abrogated in all subtypes of breast cancer. **(C)** MERTK shows significant upregulation in luminal B and TN breast cancers. The expression is not detectable in lumina A and HER2+ subtype samples. TN means triple negative.

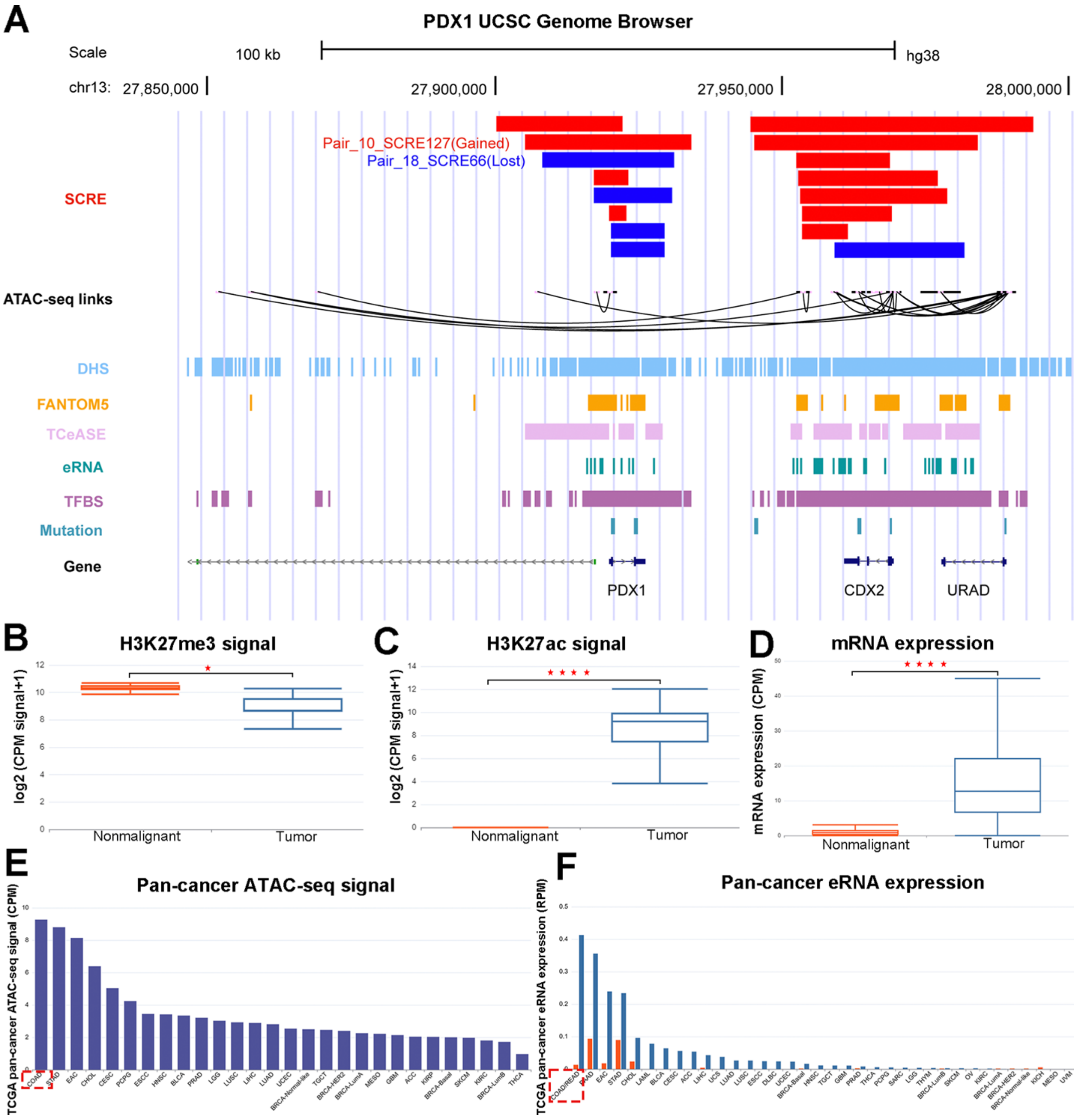

Supplementary Figure 4. An example of SCREs for PDX1. (A) Visualization of PDX1 SCREs using UCSC Genome Browser. PDX1 contains both a repressive SCRE that is lost in colorectal cancer and an active SCRE that is gained in tumors. **(B-F)** PDX1 undergoes a reduction in H3K27me3 (B) and an increase in H3K27ac (C) within SCREs, accompanied by elevated mRNA expression (D), increased accessibility (E) and enhanced eRNA activity (F) in colorectal cancer.

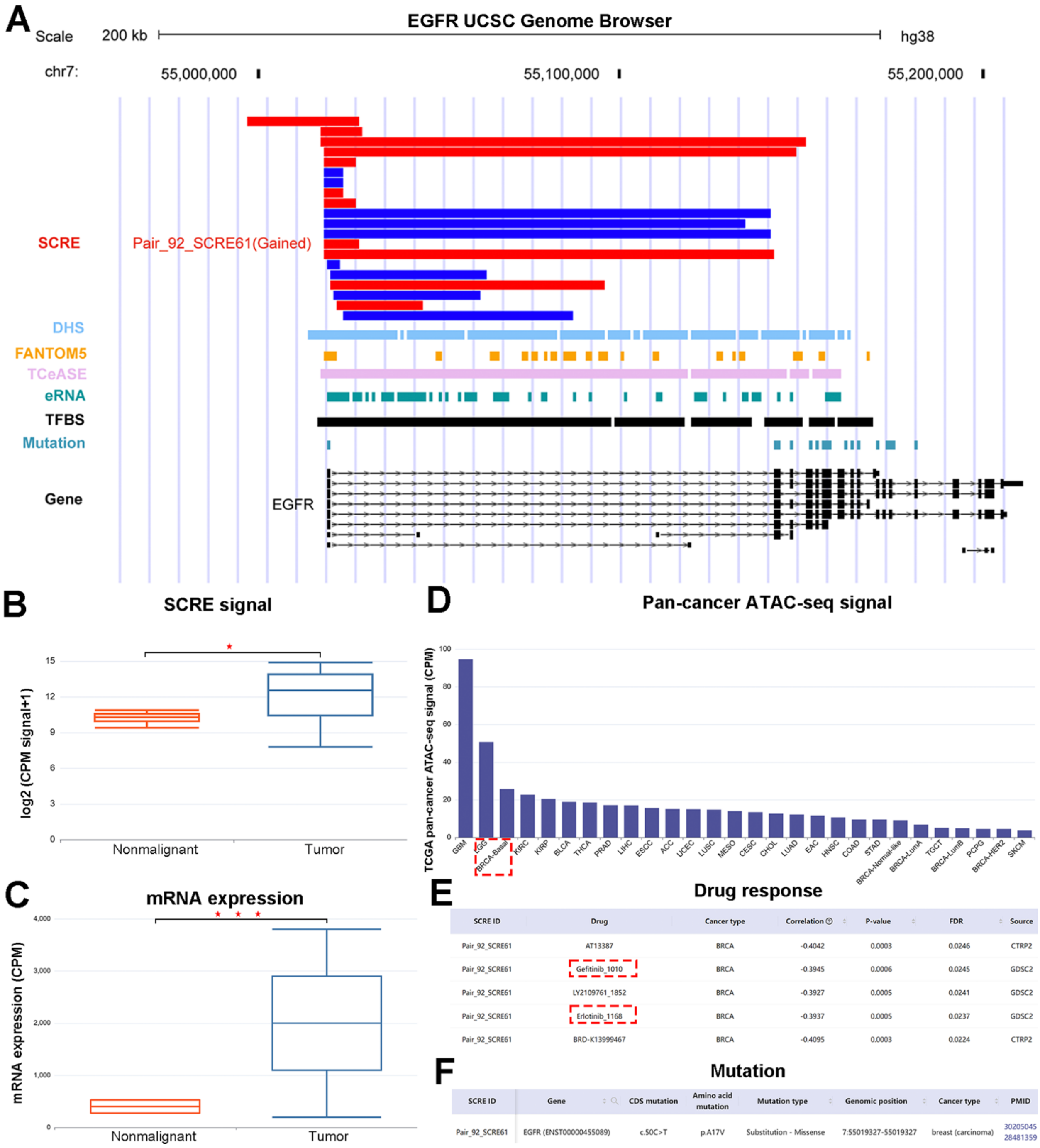

Supplementary Figure 5. An example of active SCREs for EGFR. (A) Visualization of EGFR SCREs using UCSC Genome Browser. EGFR contains an active SCRE ("Pair_92_SCRE61") that is gained in basal breast tumors. **(B-D)** EGFR shows an increase in H3K27ac within this SCRE **(B)**, elevated mRNA expression **(C)** and high accessibility **(D)** in basal breast tumors. **(E-F)** Associated drugs (E) and mutations (F) associated with this SCRE.
